# Enhanced BDNF and TrkB Activation Enhance GABA Neurotransmission in Cerebellum in Hyperammonemia

**DOI:** 10.3390/ijms231911770

**Published:** 2022-10-04

**Authors:** Yaiza M. Arenas, Mar Martínez-García, Marta Llansola, Vicente Felipo

**Affiliations:** Laboratory of Neurobiology, Centro Investigación Príncipe Felipe, 46012 Valencia, Spain

**Keywords:** hyperammonemia, cerebellum, GABAergic neurotransmission, TrkB, BDNF, neuroinflammation, Purkinje neuron, GABA_A_ receptors, motor incoordination, minimal hepatic encephalopathy

## Abstract

Background: Hyperammonemia is a main contributor to minimal hepatic encephalopathy (MHE) in cirrhotic patients. Hyperammonemic rats reproduce the motor incoordination of MHE patients, which is due to enhanced GABAergic neurotransmission in the cerebellum as a consequence of neuroinflammation. In hyperammonemic rats, neuroinflammation increases BDNF by activating the TNFR1–S1PR2–CCR2 pathway. (1) Identify mechanisms enhancing GABAergic neurotransmission in hyperammonemia; (2) assess the role of enhanced activation of TrkB; and (3) assess the role of the TNFR1–S1PR2–CCR2–BDNF pathway. In the cerebellum of hyperammonemic rats, increased BDNF levels enhance TrkB activation in Purkinje neurons, leading to increased GAD65, GAD67 and GABA levels. Enhanced TrkB activation also increases the membrane expression of the γ2, α2 and β3 subunits of GABA_A_ receptors and of KCC2. Moreover, enhanced TrkB activation in activated astrocytes increases the membrane expression of GAT3 and NKCC1. These changes are reversed by blocking TrkB or the TNFR1–SP1PR2–CCL2–CCR2–BDNF–TrkB pathway. Hyperammonemia-induced neuroinflammation increases BDNF and TrkB activation, leading to increased synthesis and extracellular GABA, and the amount of GABA_A_ receptors in the membrane and chloride gradient. These factors enhance GABAergic neurotransmission in the cerebellum. Blocking TrkB or the TNFR1–SP1PR2–CCL2–CCR2–BDNF–TrkB pathway would improve motor function in patients with hepatic encephalopathy and likely with other pathologies associated with neuroinflammation.

## 1. Introduction

Patients with liver cirrhosis may show overt or minimal hepatic encephalopathy (MHE), which affects between 30 and 70% of cirrhotic patients [1,2,3,4,5]_,_ impacting several million people around the world. Cirrhotic patients with MHE show mild cognitive impairment and motor incoordination, impaired visuomotor and bimanual coordination, impaired postural control and stability, and may show ataxia. These patients also show impairment of fine motor control; for example, they have problems with handwriting or finger tapping [6,7,8,9,10,11]. These alterations lead to increased risk of falls, fractures, and hospitalizations, and reduced driving ability, quality of life, and lifespan [12,13,14,15].

Fine motor control, postural control, and motor coordination are mainly modulated by the cerebellum [16,17,18,19,20]. Motor coordination is modulated by GABAergic neurotransmission in the cerebellum. Increased GABAergic tone in the cerebellum induces motor incoordination in rats [20,21,22,23,24]. GABAergic neurotransmission is increased in the cerebellum of patients with cirrhosis and hepatic encephalopathy (HE) [25], and in rats with chronic hyperammonemia and MHE [26]. Hassan et al. [25] used a paired-pulse transcranial magnetic stimulation paradigm in cirrhotic patients with HE and control subjects, and, on the basis of the obtained results, proposed that GABAergic tone is increased in the Purkinje neurons of patients with HE. Moreover, they showed that this increase correlated with the grade of ataxia of the patients.

Hyperammonemia is a main contributor to the neurological impairment of patients with MHE [27,28]. Rats with chronic hyperammonemia show motor incoordination which is due to increased GABAergic tone in cerebellum [26,29,30,31]. Motor coordination is restored in hyperammonemic rats by reducing GABAergic neurotransmission with pregnenolone sulphate [29], GR3027 [32], or bicuculline [33]. The intensity of GABAergic neurotransmission depends mainly on three factors: the extracellular concentration of GABA, the amount of GABA_A_ receptors present in the cell membrane surface, and the transmembrane chloride gradient [34,35].

The extracellular concentration of GABA is controlled by its synthesis by glutamate decarboxylases GAD65 and GAD67, and mainly by the action of the GABA transporters GAT3 and GAT1, which take up extracellular GABA into glial and neuronal cells, respectively [36]. The function of GAT3 and GAT1, in turn, may be rapidly modulated by changes in their amount present in the surface of the cell membrane [37]. The amount of GABA_A_ receptors in the cell membrane is modulated by regulating its trafficking [38]. Two main mechanisms involved in this regulation are through binding some GABA_A_ receptor subunits to gephyrin [39] and via phosphorylation, especially on the β3 and γ2 subunits of the GABA_A_ receptor [40,41]. The activation of the GABA_A_ receptor allows for the flow through its channel of Cl^−^, and the intensity of the response also depends on the Cl^−^ gradient across the cell membrane, which is mainly maintained by K^+^-Cl^−^ cotransporters KCC2 and NKCC1. KCC2 transports Cl^−^ out of neurons, while NKCC1 transports Cl^−^ into the cell [42,43,44]. Changes in any of the above factors may contribute to the enhanced GABAergic tone in the cerebellum of hyperammonemic rats.

The increased GABAergic tone in the cerebellum of hyperammonemic rats is a consequence of neuroinflammation. Both GABAergic tone and motor coordination are normalized by reducing neuroinflammation in the cerebellum with sulforaphane [30] or extracellular cGMP [31,45]. It is not well-known how neuroinflammation enhances GABAergic neurotransmission in the cerebellum of hyperammonemic rats. Unveiling these mechanisms may identify targets for the therapeutic treatment of motor incoordination in patients with MHE.

We showed that, in hyperammonemic rats, neuroinflammation leads to an increase in BDNF content in the cerebellum, which mainly occurs in activated microglia and is mediated by the activation of the tumor necrosis factor receptor 1 (TNFR1)–sphingosine-1-phosphate receptor 2 (S1PR2)–C-C chemokine receptor type 2 (CCR2) pathway [46]. BDNF may modulate GABAergic neurotransmission at different levels. BDNF modulates the amount in the cell membrane of GABA_A_ receptor subunits and of KCC2 [43]. BDNF also upregulates membrane expression of GABA transporter GAT1 in cultured neurons [47] and increases the transcription of GAD65 [48].

We, therefore, hypothesized that the activation of TrkB by BDNF released from activated microglia could mediate the enhanced GABAergic neurotransmission in the cerebellum of hyperammonemic rats. To advance the understanding of the mechanisms by which neuroinflammation increases the GABAergic tone in the cerebellum of hyperammonemia rats, the aims of this work are: (1) identifying the mechanisms contributing to enhanced GABAergic neurotransmission, analyzing the contribution of changes in GABA, in the membrane expression of GABA transporters, and of GABA_A_ receptor subunits and some mechanisms modulating them; (2) assessing if the enhanced activation of TrkB by BDNF is responsible for the changes in these parameters; and (3) assessing if these changes in GABAergic neurotransmission are due to the enhanced activation of the TNFR1–S1PR2–CCR2–BDNF pathway.

We analyzed the effects of hyperammonemia on the content of the cerebellar slices of GABA-synthesizing enzymes GAD65 and GAD67, and on the concentration of GABA itself, on the membrane expression of GABA transporters GAT1 and GAT3, and of GABA_A_ receptor subunits α2, β3, and γ2. We also analyzed the content of gephyrin and the phosphorylation of the β3 subunit of the GABA_A_ receptor, which are two main mechanisms modulating the membrane expression of GABA_A_ receptor subunits. To assess the role of the activation of TrkB in the effects induced by hyperammonemia on the above parameters, we assessed if these effects were reversed by blocking TrkB with the antagonist ANA12 ex vivo in cerebellar slices from hyperammonemic rats. To assess if the changes were due to enhanced activation of the TNFR1–S1PR2–CCR2 pathway, we blocked TNFR1 with R7050, S1PR2 with JTE-013, and CCR2 with RS504393.

## 2. Results

**Figure 1:**
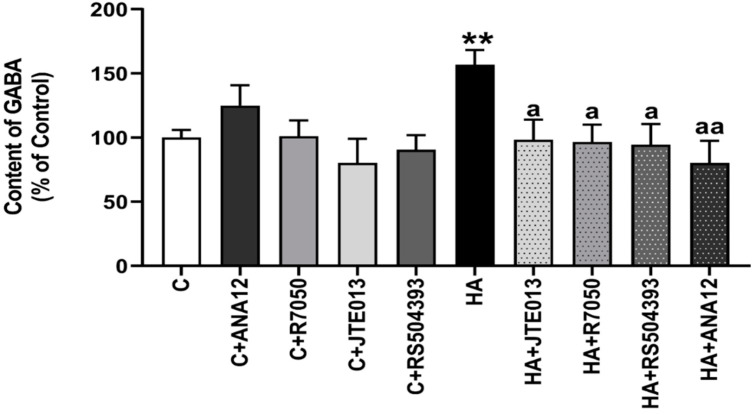
Hyperammonemia increases the content of GABA in cerebellar slices. Ex vivo treatment with ANA12, R7050, JTE-013, or RS504393 reverses the increase in GABA. HPLC was performed as indicated in Section 4. Values are the mean ± SEM of 12–16 rats per group. Values significantly different from control rats are indicated with asterisks, and from hyperammonemic rats are indicated with “a”. ** *p* < 0.01; a *p* < 0.05, aa *p* < 0.01. F = 3.628. Data were analyzed using one-way analysis of variance (ANOVA) followed by Tukey’s post hoc test.

Hyperammonemia increases (*p* < 0.009) the total content of GABA in the cerebellar slices of control rats to 157 ± 12%. This increase is completely reversed by blocking the TrkB receptor with ANA12, indicating that it is mediated by the enhanced activation of TrkB. Blocking TNFR1 signaling with R7050, S1PR2 with JTE-013, or CCR2 with RS504393 also reduced GABA levels to normal values (Figure 1). This indicates that hyperammonemia-induced neuroinflammation induces the synthesis of GABA via the TNFR1–S1PR2–CCR2–TrkB pathway.

**Figure 2:**
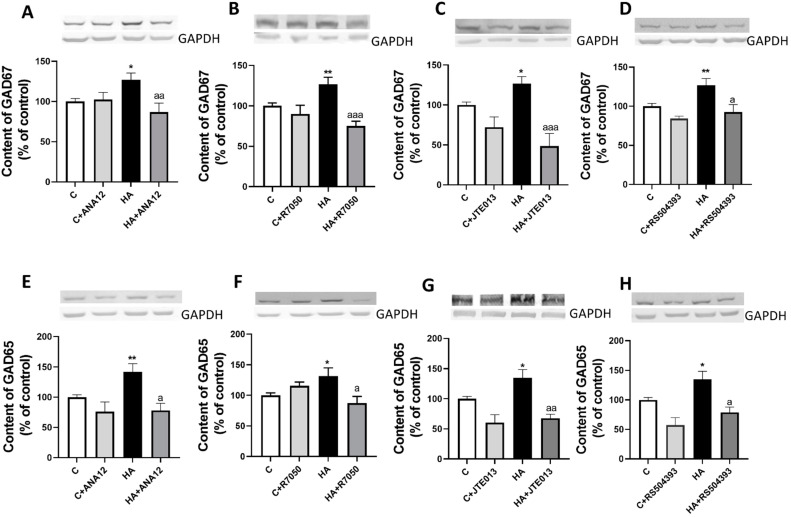
Hyperammonemia increases the content of GAD67 (**A**–**D**) and GAD65 (**E**–**H**) in cerebellar slices. Ex vivo treatment with ANA12, R7050, JTE-013, or RS504393 reverses these increases. Western blot was performed as indicated in Section 4. Values are the mean ± SEM of 19–24 rats per group. Values significantly different from control rats are indicated with asterisks, and from hyperammonemic rats are indicated with “a”. * *p* < 0.05; ** *p* < 0.01; a *p* < 0.05, aa *p* < 0.01; aaa *p* < 0.001. (**A**–**H**) F values were 4.450, 7.968, 9.863, 5.499, 6.864, 3.683, 7.582, and 7.051, respectively. Data were analyzed using one-way analysis of variance (ANOVA) followed by Tukey’s post hoc test.

The main enzymes responsible for GABA synthesis in the cerebellum are GAD67 and GAD65. We, therefore, analyzed if hyperammonemia increases the content of GAD67 and/or GAD65. As shown in Figure 2, the content of GAD67 increased in the slices from hyperammonemic rats to 127 ± 8% of the control rats, as assessed with Western blot. This increase was completely reversed by blocking TrkB with ANA12 (Figure 2A), TNFR1 signaling with R7050 (Figure 2B), S1PR2 with JTE-013 (Figure 2C), or CCR2 with RS504393 (Figure 2D).

**Figure 3:**
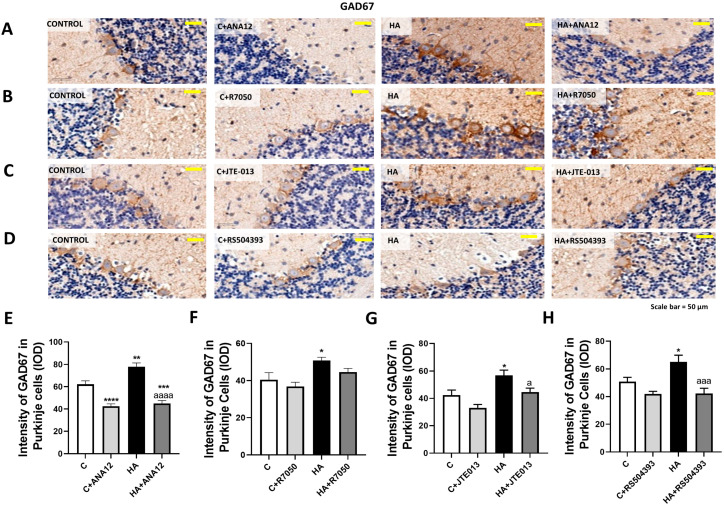
GAD67 content increased in the Purkinje neurons of hyperammonemic rats. Ex vivo treatment with ANA12, R7050, JTE-013, or RS504393 reversed this increase. (**A**–**D**) Immunohistochemistry was performed as indicated in Section 4, with DAB staining using antibodies against GAD67. Representative images are shown. (**E**–**H**) GAD67 content was quantified in Purkinje cells as described in Section 4. Values are the mean ± SEM of 8 rats per group. Values significantly different from control rats are indicated with asterisks, and from hyperammonemic rats are indicated with “a”. * *p* < 0.05, ** *p* < 0.01, *** *p* < 0.001, **** *p* < 0.0001; a *p* < 0.05, aaa *p* < 0.001, aaaa *p* < 0.0001. (**E**–**H**) F values were 35.26, 5.622, 9.090, and 8.133, -respectively. Data were analyzed using one-way analysis of variance (ANOVA) followed by Tukey’s post hoc test.

GAD67 especially increased in Purkinje cells to 62 ± 3% (*p* < 0.001) of the control rats, as assessed with immunohistochemistry (Figure 3), and this increase was also reversed by blocking TrkB (Figure 3A,E), TNFR1 signaling (Figure 3B,F), S1PR2 (Figure 3C,G), or CCR2 (Figure 3D,H).

We also analyzed if hyperammonemia increases the content of GAD65. As shown in Figure 2, the content of GAD65 increased (*p* < 0.006) in slices from hyperammonemic rats to 142 ± 13% of the control rats, as assessed with Western blot. This increase was completely reversed by blocking TrkB receptor with ANA12 (Figure 2E), TNFR1 signaling with R7050 (Figure 2F), S1PR2 with JTE-013 (Figure 2G), or CCR2 with RS504393 (Figure 2H).

**Figure 4:**
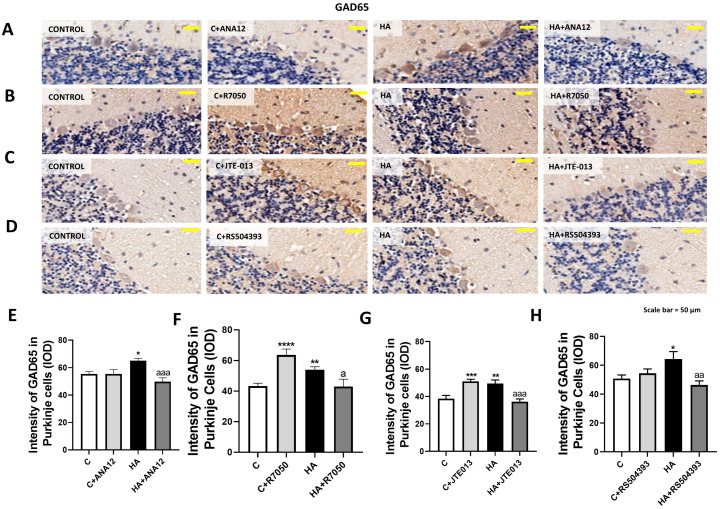
GAD65 content increased in the Purkinje neurons of hyperammonemic rats. Ex vivo treatment with ANA12, R7050, JTE-013, or RS504393 reversed this increase. (**A**–**D**) Immunohistochemistry was performed as indicated in Section 4 with DAB staining using antibodies against GAD65. Representative images are shown. (**E**–**H**) GAD67 content was quantified in Purkinje cells as described in Section 4. Values are the mean ± SEM of 8 rats per group. Values significantly different from control rats are indicated with asterisks, and from hyperammonemic rats are indicated by “a”. * *p* < 0.05, ** *p* < 0.01, *** *p* < 0.001; **** *p* < 0.0001; a *p* < 0.05, aa *p* < 0.01, aaa *p* < 0.001. (**E**–**H**) F values were 6.688, 10.52, 13.14, and 4.657, respectively. Data were analyzed using one-way analysis of variance (ANOVA) followed by Tukey’s post hoc test.

GAD65 increased in Purkinje neurons to 59 ± 2% (*p* < 0.005) of the control rats, as assessed with immunohistochemistry (Figure 4), which was also reversed by blocking TrkB (Figure 4A,E), TNFR1 signaling (Figure 4B,F), S1PR2 (Figure 4C,G), or CCR2 (Figure 4D,H).

The above data show that hyperammonemia increases the content of GAD67 and GAD65, and the synthesis of GABA in the cerebellum, especially in Purkinje neurons. This would contribute to the enhanced GABAergic tone in the cerebellum. However, the intensity of GABA responses also depends on other parameters, such as:

(1) The extracellular concentration of GABA, as only extracellular GABA may activate GABA_A_ receptors in the cell membrane.
(2) The amount of GABA_A_ receptors in the cell membrane.

We, therefore, analyzed the main aspects modulating the above parameters. Extracellular GABA levels are mainly modulated by GABA transporters GAT1 and GAT3. Extracellular GABA concentration is increased in the cerebellum of hyperammonemic rats [26,30,31], and may be due to the enhanced membrane expression and reversed function of GAT3 in activated astrocytes [30]. However, the underlying mechanisms have not been studied.

**Figure 5:**
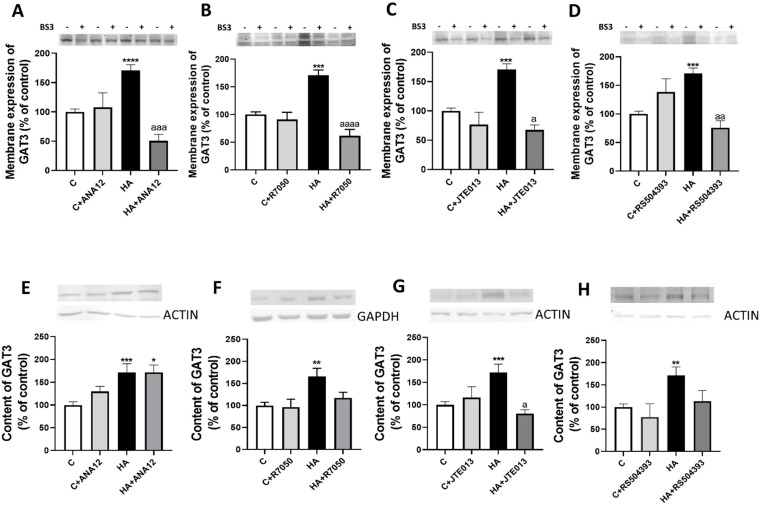
Hyperammonemia increases the membrane expression and total content of GAT3. Ex vivo treatment with ANA12, R7050, JTE-013 or RS504393 reverses most of these increases. (**A**–**D**) Membrane expression of GAT3 was analyzed using the BS3 cross-linker procedure in slices from control (C) and hyperammonemic (HA) rats. The effects of blocking TrkB with TrkB with ANA12, TNFR1 with R7050, S1PR2 with JTE-013, and CCR2 with RS504393 on the membrane expression of GAT3 are also shown. (**E**–**H**) Total GAT3 content was analyzed with Western blot in slices from control and hyperammonemic rats treated or not with ANA12, R7050, JTE-013, and RS504393. Actin or GAPDH was used as the loading control. Values are the mean ± SEM of (**A**–**D**) 23–32 rats and (**E**–**H**) 17–25 rats per group. Values significantly different from control rats are indicated with asterisks, and from hyperammonemic rats are indicated with “a”. * *p* < 0.05, ** *p* < 0.01, *** *p* < 0.001, **** *p* < 0.0001; a *p* < 0.05, aa *p* < 0.01, aaa *p* < 0.001, aaaa *p* < 0.0001. (**A**–**H**) F values were 8.691, 14.13, 8.873, 7.979, 7.813, 5.912, 6.499, and 6.537, respectively. Data were analyzed using one-way analysis of variance (ANOVA) followed by Tukey’s post hoc test.

We assessed whether the activation of TrkB by BDNF is responsible for the increased membrane expression of GAT3 and GAT1. The membrane expression of GAT3 is increased in the cerebellum of hyperammonemic rats, and blocking TrkB with ANA12 completely reverses this increase (Figure 5A). A similar normalization was obtained by blocking TNFR1 (Figure 5B), S1PR2 (Figure 5C), or CCR2 (Figure 5D).

We also analyzed if hyperammonemia affects the content of GAT3 in the cerebellum. As shown in Figure 5, GAT3 content increased (*p* < 0.001) in the cerebellum of hyperammonemic rats. This increase was not reversed by blocking TrkB with ANA12 (Figure 5E), but was completely reversed by blocking TNFR1 (Figure 5F), S1PR2 (Figure 5G), or CCR2 (Figure 5H).

**Figure 6:**
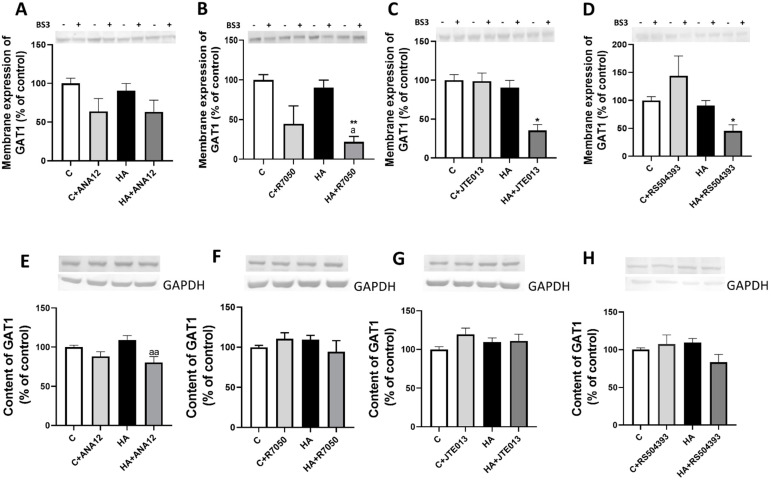
Hyperammonemia does not affect the membrane expression and content of GAT1. (**A**–**D**) The membrane expression of GAT1 was analyzed using a BS3 cross-linker procedure in slices from control (C) and hyperammonemic (HA) rats. The effects of blocking TrkB with ANA12, TNFR1 with R7050, S1PR2 with JTE-013, and CCR2 with RS504393 on the membrane expression of GAT1 are also shown. (**E**–**H**) Total content of GAT1 was analyzed with Western blot in slices from control and hyperammonemic rats treated or not with ANA12, R7050, JTE-013, and RS504393. Actin or GAPDH were used as the loading control. Values are the mean ± SEM of (**A**–**D**) 23–32 rats and (**E**–**H**) 17–25 rats per group. Values significantly different from control rats are indicated with asterisks, and from hyperammonemic rats are indicated with “a”. * *p* < 0.05, ** *p* < 0.01; a *p* < 0.05, aa *p* < 0.01. (**A**–**H**) F values were 2.681, 6.521, 2.793, 5.908, 5.711, 1.371, 1.371, 1.536, and 2.566, respectively. Data were analyzed using one-way analysis of variance (ANOVA) followed by Tukey’s post hoc test.

The membrane expression of GAT1 was not altered (*p* < 0.8) in the cerebellum of hyperammonemic rats and blocking TrkB receptor with ANA12 (Figure 6A), TNFR1 signaling with R7050 (Figure 6B), S1PR2 with JTE-013 (Figure 6C) or CCR2 with RS504393 (Figure 6D) reduced membrane expression of GAT1.

We also analyzed if hyperammonemia affects the content of GAT1 in cerebellum. As shown in Figure 6E–H, GAT1 content was not altered in cerebellum by hyperammonemia nor by any of the treatments.

The response to GABA_A_ receptor activation by extracellular GABA also depends on the amount of GABA_A_ receptors in the cell membrane. We, therefore, analyzed the membrane expression of the main GABA_A_ receptor subunits in the cerebellum.

**Figure 7:**
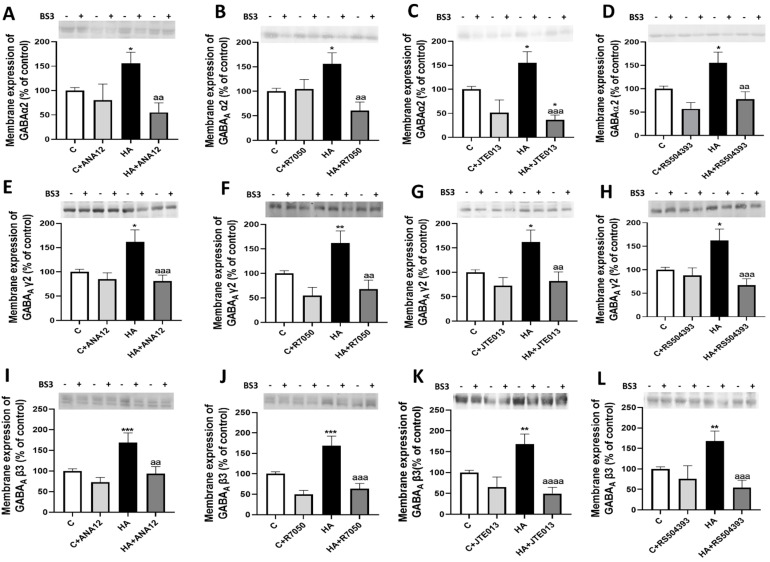
Hyperammonemia increases the membrane expression of GABAA receptor subunits α2, γ2, and β3 in the cerebellum. Ex vivo treatment with ANA12, R7050, JTE-013, or RS504393 reverses these increases. The membrane expression of GABAA (**A**–**D**) α2, (**E**–**H**) γ2, and (**I**–**L**) β3 in the cerebellum was analyzed using BS3 cross-linker procedure in slices from control (C) and hyperammonemic (HA) rats. The effects of blocking TrkB with ANA12, TNFR1 with R7050, S1PR2 with JTE-013, and CCR2 with RS504393 on membrane expression of this subunits of GABAA receptor are also shown. Values are the mean ± SEM of 15–24 rats per group. Values significantly different from control rats are indicated with asterisks, and from hyperammonemic rats are indicated with “a”. * *p* < 0.05, ** *p* < 0.01, *** *p* < 0.001; aa *p* < 0.01, aaa *p* < 0.001, aaaa *p* < 0.0001. (**A**–**L**) F values were 5.761, 5.959, 10.34, 8.885, 6.042, 7.936, 5.989, 6.573, 8.996, 12.06, 10.77, and 8.563, respectively. Data were analyzed using one-way analysis of variance (ANOVA) followed by Tukey’s post hoc test.

Hyperammonemia increased the membrane expression of the GABA γ2, α2, and β3 subunits (Figure 7), and did not affect the membrane expression of the α1, α6, and δ subunits (not shown). The increases in membrane expression of the γ2, (Figure 7A–D), α2 (Figure 7E–H), and β3 (Figure 7I–L) subunits were completely reversed by blocking TrkB with ANA12, TNFR1 with R7050, S1PR2 with JTE-013, or CCR2 with RS504393 (Figure 7A–L), indicating that they are mediated by the enhanced activation of TrkB by BDNF as a consequence of the increased activation of the TNFR1–S1PR2–CCR2 pathway.

**Figure 8:**
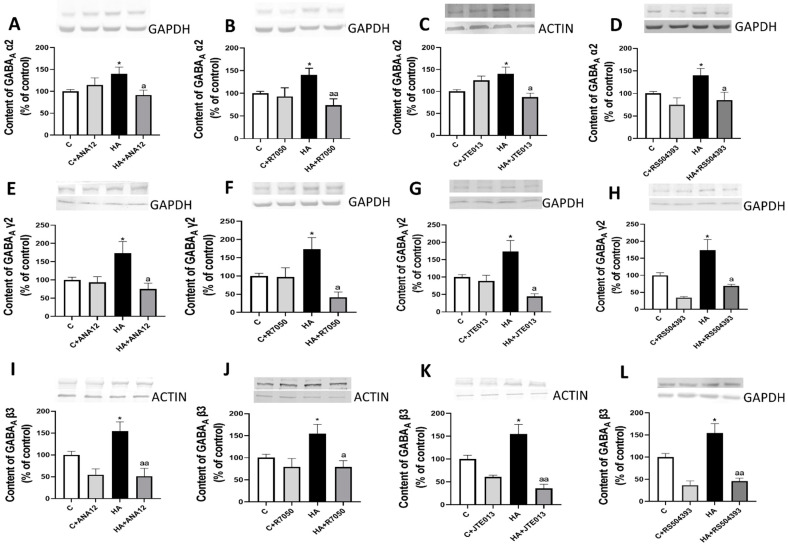
Hyperammonemia increases the content of GABAA receptor subunits α2, γ2, and β3 in the cerebellum. Ex vivo treatment with ANA12, R7050, JTE-013, or RS504393 reverses these increases. The total content of GABAA receptor subunits (**A**–**D**) α2, (**E**–**H**) γ2, and (**I**–**L**) β3 in the cerebellum of control (C) and hyperammonemic (HA) rats was analyzed with Western blot. The effects of blocking TrkB with ANA12, TNFR1 with R7050, S1PR2 with JTE-013, or CCR2 with RS504393 on the content are also shown. Values are the mean ± SEM of 18–26 rats per group. Values significantly different from control rats are indicated with asterisks, and from hyperammonemic rats are indicated with “a”. * *p* < 0.05; a *p* < 0.05, aa *p* < 0.01. (**A**–**L**) F values were 3.825, 5.274, 4.670, 5.303, 3.753, 3.720, 3.805, 5.340, 7.284, 4.487, 6.564, and 7.261, respectively. Data were analyzed using one-way analysis of variance (ANOVA) followed by Tukey’s post hoc test.

Similar results were obtained for the total content of the GABA_A_ receptor subunits in whole homogenates of cerebellar slices. Hyperammonemia increased the content of the GABA γ2 (Figure 8A–D), α2 (Figure 8E–H), and β3 (Figure 8I–L) subunits, which were completely reversed by blocking TrkB with ANA12, TNFR1 with R7050, S1PR2 with JTE-013, or CCR2 with RS504393 (Figure 8A–L).

**Figure 9:**
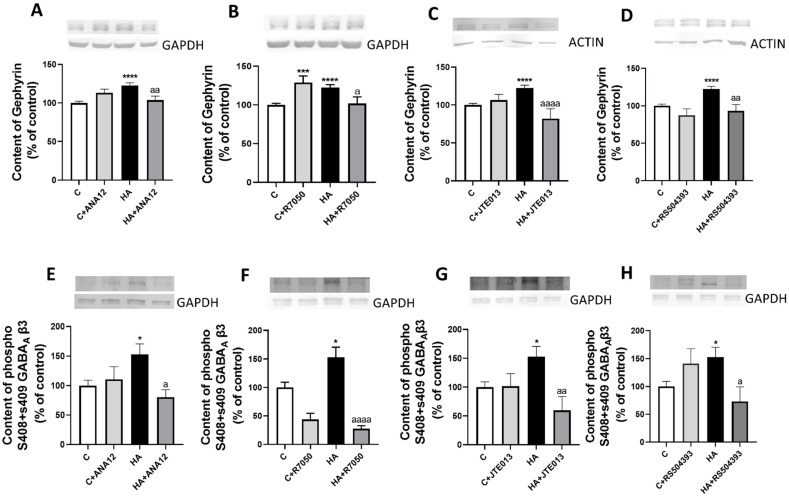
Hyperammonemia increases the content of gephyrin and the phosphorylation of the β3 subunit of the GABAA receptor in the cerebellum. This is reversed by blocking TrkB, TNFR1, S1PR2 or CCR2. (**A**–**D**) Gephyrin content and (**E**–**H**) the phosphorylation of the β3 subunit of the GABAA receptor in the cerebellum of control (C) and hyperammonemic (HA) rats were analyzed with Western blot. The effects of blocking TrkB with ANA12, TNFR1 with R7050, S1PR2 with JTE-013, or CCR2 with RS504393 on the content are also shown. Values are the mean ± SEM of 15–20 rats per group. Values significantly different from control rats are indicated with asterisks, and from hyperammonemic rats are indicated with “a”. * *p* < 0.05, *** *p* < 0.001, **** *p* < 0.0001; a *p* < 0.05, aa *p* < 0.01, aaaa *p* < 0.0001. (**A**–**H**) F values were 10.13, 12.54, 13.19, 14.37, 4.059, 12.25, 4.927, and 4.183, respectively. Data were analyzed using one-way analysis of variance (ANOVA) followed by Tukey’s post hoc test.

Two main mechanisms modulating the membrane expression of GABA_A_ receptor subunits are the binding to gephyrin and phosphorylation of the β3 subunit. Hyperammonemia increased the content of gephyrin (Figure 9A–D) and the phosphorylation of the β3 subunit of GABA_A_ receptor (Figure 9E–H) in the cerebellum; these increases were reversed by blocking TrkB with ANA12, TNFR1 with R7050, S1PR2 with JTE-013, or CCR2 with RS504393.

## 3. Discussion

**Figure 10:**
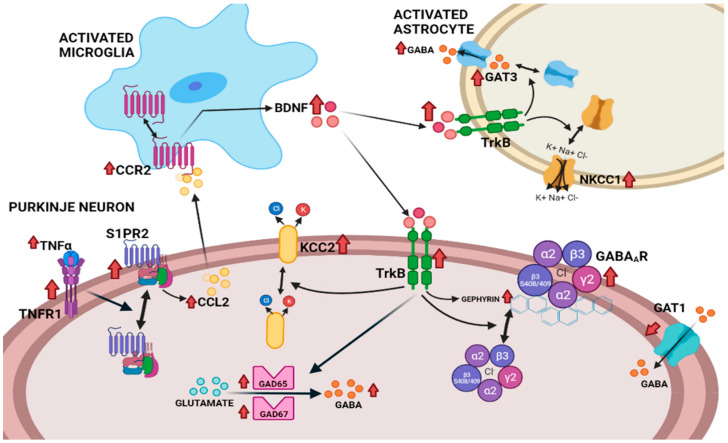
Proposed pathway by which the neuroinflammation-induced enhanced activation of the TNFR1–S1PR2–CCL2–CCR2–BDNF–TrkB pathway enhances GABAergic neurotransmission in the cerebellum of hyperammonemic rats. Hyperammonemia-induced neuroinflammation increases the levels of BDNF in microglia through the activation of the TNFR1–S1PR2–CCL2–CCR2 pathway. Increased levels of BDNF enhance the activation of TrkB in Purkinje neurons, leading to increased levels of GABA-synthesizing enzymes GAD65 and GAD67, and of GABA. Enhanced TrkB activation also increases the membrane expression of the γ2, α2, and β3 subunits of GABAA receptors and of KCC2. Moreover, the enhanced activation of TrkB in activated astrocytes increases the membrane expression of GAT3 and of NKCC1, which also contributes to enhanced extracellular GABA. The increase in extracellular GABA and the membrane expression of GABAA receptors, and of KCC2 in Purkinje neurons results in enhanced GABAergic neurotransmission. All these changes in GABAergic neurotransmission are reversed by blocking TrkB or the TNFR1–SP1PR2–CCL2–CCR2–BDNF–TrkB pathway at any of its steps. The effects of hyperammonemia are indicated with red arrows (↑).

This report identifies several mechanisms by which a neuroinflammation-induced increase in BDNF enhances GABAergic neurotransmission in the cerebellum of hyperammonemic rats. These mechanisms are summarized in Figure 10. The enhanced activation of TrkB by BDNF affects several aspects of GABAergic neurotransmission, mainly in Purkinje neurons. The main effects induced by the increased TrkB activation in the cerebellum of hyperammonemic rats are: (1) the increased content of GABA-synthesizing enzymes GAD65 and GAD67, which results in the increased synthesis of GABA that contributes to its increased extracellular concentration; (2) the increased membrane expression of GAT3 in activated astrocytes, which also contributes to enhanced extracellular GABA concentration, as discussed below; (3) the increased membrane expression of GABA_A_ receptors, which is mediated by increased levels of gephyrin, and of the phosphorylation of the β3 subunit of GABA_A_ receptors. These effects are mediated by the activation of TrkB by the enhanced levels of BDNF, as supported by the fact that all these effects are reversed by blocking TrkB with ANA12. Moreover, most of these effects seem to occur mainly in Purkinje neurons and result in the enhanced GABAergic neurotransmission in the cerebellum of hyperammonemic rats [26,29,30,31]. It is very likely that these mechanisms are also responsible for the enhanced GABAergic tone in the Purkinje neurons of cirrhotic patients with hepatic encephalopathy, as reported by Hassan et al. [25].

The selective effect on Purkinje neurons agrees with the report of Cheng and Yeh [49], who showed that the activation of TrkB by BDNF in cerebellar slices enhances GABA_A_-receptor-mediated currents in Purkinje neurons, but reduces them in granular neurons. The authors suggested that the potentiation of GABA_A_ receptor responses by TrB activation in Purkinje neurons is due to the increased phosphorylation and/or surface expression of GABA_A_ receptors. A modulation of the membrane expression of GABA_A_ receptors by BDNF was also reported by Jovanovic et al. [50]. We show here that, in the cerebellum of hyperammonemic rats, the enhanced activation of TrkB via increased BDNF levels increases the membrane expression of the γ2, α2, and β3 subunits of GABA_A_ receptors. These data and the above reports suggest that this increase mainly occurs in Purkinje neurons.

The reported results also suggest that the increased membrane expression of GABA_A_ receptors in hyperammonemia is mediated by the BDNF–TrkB-induced increase in gephyrin and in the phosphorylation of the β3 subunit of GABA_A_ receptors. The amount of GABA_A_ receptors in the cell membrane is modulated by regulating its trafficking [38]. Two main mechanisms involved in this regulation are by binding some GABA_A_ receptor subunits to gephyrin [39] and by phosphorylation, especially on the β3 and γ2 subunits of the GABA_A_ receptor [40,41].

Gephyrin binds to the alpha subunits of GABA_A_ receptors, promoting their clustering [39,51]. Gephyrin content increases in hyperammonemic rats, which contributes to the enhanced membrane expression of GABA_A_ receptors.

Binding protein AP2 to GABA_A_ receptor subunits β3 and γ2 promotes the endocytosis of GABA_A_ receptors. The phosphorylation of the β3 subunit at Serines 408–409 prevents AP2 binding, thus reducing the endocytosis of GABA_A_ receptors and increasing their content in the membrane [40]. The increased phosphorylation of the β3 subunit in hyperammonemic rats prevents endocytosis and contributes to the increased membrane expression of GABA_A_ receptors, which in turn contributes to enhanced GABAergic neurotransmission.

The increase in the content of GAD65 and GAD67 also occurs in Purkinje neurons, which contributes to the increased synthesis and extracellular concentration of GABA.

Another main contributor to the increased levels of extracellular GABA is the BDNF–TrkB-induced increase in GAT3, which mainly occurs in activated astrocytes. In activated astrocytes, the function of GAT3 is reversed: instead of taking up GABA from the extracellular space into the astrocytes, it releases GABA from the inside to the outside of the astrocyte [52,53,54,55]. Here, blocking TrkB reduced the membrane expression of GAT3, thus normalizing the uptake of extracellular GABA by astrocytes, which contributes to reducing extracellular GABA to the normal levels, as previously shown in hyperammonemic rats treated with sulforaphane or extracellular cGMP to reduce neuroinflammation [30,31].

Another mechanism by which the enhanced activation of TrkB enhances GABAergic neurotransmission in hyperammonemic rats is by altering the membrane expression of K^+^-Cl^−^ cotransporters KCC2 and NKCC1. The activation of GABA_A_ receptor allows for Cl^−^ flow through its channel, and the intensity of the response also depends on the Cl^−^ gradient across the cell membrane, which is mainly maintained by K^+^-Cl^−^ cotransporters KCC2 and NKCC1. KCC2 transports Cl^−^ out of neurons, while NKCC1 transports Cl^−^ into the cell [42,43,44,56,57]. We recently showed that chronic hyperammonemia increases KCC2 and NKCC1 membrane expression in the cerebellum, and that blocking TrkB reverses these effects [46]. The increased membrane expression of KCC2 and NKCC1 contributes to enhance GABAergic neurotransmission in the cerebellum and induces motor incoordination in hyperammonemic rats [46]. The increased membrane expression of KCC2, which is expressed especially in Purkinje neurons [56,57], increases the extrusion of Cl^−^ from Purkinje neurons, thus increasing the Cl^−^ gradient through the membrane and thereby the intensity of GABA responses in Purkinje neurons. Blocking TrkB with ANA12 normalizes the membrane expression of KCC2, which normalizes the Cl^−^ gradient in Purkinje neurons, thus reducing the GABA responses to normal levels [46].

Hyperammonemia also increases the membrane expression of NKCC1, which is mainly present in astrocytes and granular neurons [56,58]. This enhances the uptake of Cl^−^ by NKCC1 in these cell types, which reduces the Cl^−^ gradient through the membrane, and thereby the intensity of the GABA responses in astrocytes and granular neurons. Blocking TrkB with ANA12 also normalizes the membrane expression of NKCC1, which normalizes the Cl^−^ gradient and GABA responses in these cell types [46].

We also analyzed if the increased levels of BDNF and TrkB activation were due to the enhanced activation of the TNFR1–SP1PR2–CCL2 (monocyte chemoattractant protein-1)–CCR2–BDNF–TrkB pathway in hyperammonemia (Figure 10). We recently showed that, in the cerebellum of hyperammonemic rats, the enhanced activation of TNFR1 increases the activation of S1PR2, which induces an increase in CCL2 levels in Purkinje neurons, which activates CCR2 in the microglia, leading to microglia activation and increased levels of BDNF in the microglia [46]. The increase in BDNF levels was reversed by blocking TNFR1 signaling with R7050, S1PR2 with JTE-013, or CCR2 with RS504393. We show here that the hyperammonemia-induced changes in GAD65 and GAD67, and in the membrane expression of GAT3, and of the γ2, α2, and β3 subunits of GABA_A_ receptors are also reversed by blocking TNFR1 signaling, S1PR2, or CCR2. This indicates that the increased function of the TNFR1–SP1PR2–CCL2–CCR2–BDNF pathway in the cerebellum of hyperammonemic rats is responsible for the enhanced activation of TrkB and the subsequent effects on these parameters of GABAergic neurotransmission (Figure 10). This suggests that reducing the function of the TNFR1–SP1PR2–CCL2–CCR2–BDNF pathway at any of its steps reduces BDNF levels and reverse alterations in GABAergic neurotransmission in hyperammonemia and likely in patients with hepatic encephalopathy. This opens new therapeutic possibilities to improve motor coordination and cognitive function in these patients.

There is increasing evidence that neuroinflammation is a main contributor to the cognitive and motor alterations in many neurological and neurodegenerative diseases, including multiple sclerosis [59], Alzheimer’s disease [60], Parkinson’s disease [61], Huntington’s disease [62], autism [63], and hyperammonemia and hepatic encephalopathy [45]. The motor and cognitive impairment induced by neuroinflammation is mediated by alterations in neurotransmission. Neuroinflammation alters glutamatergic and GABAergic neurotransmission, but the underlying mechanisms remain unclear [45]. Altered GABAergic neurotransmission in the cerebellum plays a relevant role in the pathogenesis of autism [64], hyperammonemia, and hepatic encephalopathy [25,26,45], and is also involved in Huntington’s disease [65], Alzheimer’s disease [66,67], Parkinson’s disease, and multiple sclerosis [68].

## 4. Materials and Methods

Male Wistar rats (220–250 g) were made hyperammonemic by feeding them a diet containing ammonium acetate for 4–5 weeks, as in the study by Felipo et al. [69], and modified to contain 25% ammonium acetate, as in the study by Taoro-Gonzalez et al. [70] Three rats were housed per cage (425 × 266 × 185 mm from Tecniplast, Buguggiate, Italy) with ad libitum access to food and water. Animals were kept under a controlled temperature (23 ± 1 °C), humidity (55 ± 5%), and a 12/12 h light/dark cycle. The experiments were approved by the Comite de Experimentación y Bienestar Animal (CEBA) of our center and by the Conselleria de Agricultura of Generalitat Valenciana, and were performed in accordance with the guidelines of the Directive of the European Commission (2010/63/EU) for the care and management of experimental animals. The total number of animals was 32 for the control, and 32 for the hyperammonemic rats.

### 4.1. Experimental Design

We analyzed the content, phosphorylation, and/or membrane expression of different proteins in freshly isolated cerebellar slices from control and hyperammonemic rats (see below). The role of TrkB was assessed by blocking it via adding antagonist ANA12 to the slices (TOCRIS Bioscience cat. no. 4781); the role of TNFR1 by blocking the formation of the TNFR1–TRADD/RIP1/TRAF2 complex by adding R7050 (TOCRIS Bioscience cat. no. 5432), the role of S1PR2 by blocking it with JTE013 (TOCRIS Bioscience cat. no. 2392), and the role of CCR2 by blocking it with RS504393 (TOCRIS Bioscience cat. no. 2517). After incubation for 30 min, we examined if these treatments affected the content, phosphorylation, and/or membrane expression of the studied proteins, and neuroinflammation via immunochemistry.

### 4.2. Analysis of Protein Content and Phosphorylation in Cerebellar Slices with Western Blot

Control and hyperammonemic rats were sacrificed at 4–5 weeks of hyperammonemia. The cerebellum was immersed in a Krebs buffer simulating cerebrospinal fluid (119 mM NaCl, 26.2 mM NaHCO_3_, 11 mM glucose, 2.5 mN KCl, 2.5 mM CaCl_2_, 1 mM KH_2_PO_4_), on ice, and bubbled with carbogen (95% O_2_ and CO_2_ 5%) to preserve the oxygenated tissue and reduce necrosis. Cerebellar slices (400 μm thick, transversal) were cut, and 50 μM ANA12 to block TrkB [71,72], 20 μM R7050 to block the formation of the complex TNFR1-TRADD/RIP1/TRAF2 [73], 20 μM JTE-013, an antagonist of the S1PR2 receptor [74], or 50 μM RS504393 to block CCR2 [75] was added to the slices and incubated for 30 min. Samples were subjected to immunoblotting as in [76], using actin or GAPDH as a control for protein loading and using antibodies against GAT1 (1:500, ab426), GAT3 (1:500, ab431), GABAγ2 (1:500, ab16213), GABAβ3 (1:500, ab98968), GAD67 (1:1000, ab26116), gephyrin (1:1000, ab181382) all from Abcam (Cambridge, UK), GABAα2 (1:1000, BS-12061R) from Bioss Antibodies Inc. (Woburn, MA, USA), GAD65 (1:1000, G-1166) from SIGMA (St. Louis, MO, USA), and pSer408/409-GABA β3 (1:500, p1130-4089) from PHOSPHO SOLUTIONS (Denver, CO, USA). As a control for protein loading, the same membranes used to quantify the amount of proteins were incubated with an antibody against actin (1:5000, ab6276) from Abcam (Cambridge, UK), or GAPDH (1:10000, MAB374) from Millipore (Burlington, MA, USA), depending on the molecular mass of the analyzed proteins. Secondary antibodies were antirabbit (cat. no. A8025), antigoat (cat. no. A7650), or antimouse (cat. no. A3562) IgG, 1:4000 dilution conjugated with alkaline phosphatase from SIGMA (St. Louis, MO, USA). The images were captured using a ScanJet 5300C (Hewlett-Packard, Amsterdam, The Netherlands), and band intensities were quantified using an Alpha Imager 2200, version 3.1.2 (AlphaInnotech Corporation, San Francisco, CA, USA).

### 4.3. Analysis of Membrane Expression of Different Subunits of GABA_A_R and Other Proteins

The membrane expression of proteins in cerebellar slices was analyzed with cross-linking with BS3 (Pierce cat. no. 21580, Rockford, IL, USA), as described by Cabrera-Pastor et al. [77] After the treatments (see above), slices were added to tubes containing ice-cold Krebs buffer with or without 2 mM BS3 and incubated for 30 min at 4 °C with gentle shacking. Cross-linking was terminated by quenching the reaction with 100 mM glycine (10 min, 4 °C). The slices were homogenized by sonication for 20 s. Samples treated or not with BS3 were analyzed with Western blot, as described above, using antibodies GAT1 (1:500), GAT3 (1:500), GABAγ2 (1:500), GABAβ3 (1:500) from Abcam, and GABAα2 (1:1000) from BIOSS Antibodies. The membrane surface expression of each subunit was calculated as the difference between the intensity of the bands without BS3 (total protein) and with BS3 (nonmembrane protein).

### 4.4. Immunohistochemistry

After the incubation of cerebellar slices with different treatments, as described above, slices were fixed in 4% paraformaldehyde in 0.1 M phosphate buffer (pH 7.4) for 24 h at 4 °C. Paraffin-embedded slices were incubated with antibodies against GAD65 (SIGMA; 1:200) and GAD67 (ABCAM 1:1000) overnight. Then, slides were incubated with goat antimouse (HRP polymer) secondary antibody (Abcam) for 1 h, and diaminobenzidine for 10 min. Sections were counterstained with Mayer’s hematoxylin (DAKO) for 5 min. Once the slides had dried, they were scanned using an Aperio Versa scanner (Leica Biosystems, Nussloch, Germany). Scanned slides were analyzed using ImageScope64 software, which allows for photos of areas of interest to be obtained at different magnifications. In all immunohistochemistry analyses, we include a control without a primary antibody where no signal was seen, indicating that the staining reflects the true binding of the antibodies.

### 4.5. Analysis of GAD65 and GAD67 Content in Purkinje Neurons

The analysis of staining of each protein was performed in Purkinje neurons using Image J software. Purkinje neurons were manually selected using the freehand selection of the ROI manager function, and the mean intensities (M.I.) of staining for GAD65 and GAD67 were recorded. The analysis was performed on at least 10 40× fields for each rat.

### 4.6. Determination of GABA in Cerebellum Slices with Liquid Chromatography–Mass Spectrometry (HPLC–MS)

The concentrations of GABA in the cerebellar slices treated ex vivo with the different treatments were measured with HPLC–MS. The slices were homogenized in 100 µL of H_2_O (specific water for HPLC) on ice, sonicating twice for 20 s each time; 20 µL of trifluoroacetic acid was added in a hood to precipitate the proteins, and they were centrifuged at 20,000× *g* for 15 min at 4 °C. The supernatant was then transferred to another tube to load the 96-well LC–MS plate with 40 µL of the sample. In the pellet, 100 µL of 0.5M NaOH was added and dissolved, and 5 µL was taken to measure the protein concentration.

Chromatography was performed on a high-performance liquid chromatography (HPLC) (LC EXION, AB Sciex, Old Connecticut Path, Framingham, MA, USA) system with an Atlantis HILIC Silica column (3.0 microns i.d., 100 × 2.1 mm) (Waters, Milford, MA, USA). The mobile phase contained 0.1% formic acid in water (A) and 0.1% formic acid in acetonitrile (B). The gradient program was: 90% A 0–1.5 min, 15% A at 1.7 min, 15% A 3 min; 90% at 3.1 min and 90% at 4.5 min. The flow rate was 0.4 mL/min; the column temperature 30 °C, and the injection volume 30 μL. The HPLC was coupled to a QTRAP 4500 triple quadrupole mass spectrometer (AB Sciex) equipped with an electrospray ionization (ESI) ion source that was used in positive ion mode. The conditions were the following: inlet potential 10, curtain gas 20, ungrouping potential 46 V, Collision energy 15 eV, GAS1 40 and GAS2 30, 600 °C and 4500 V in multiple-reaction monitoring mode (MRM) with the following transition for the quantification of glutamate: 148 *m*/*z* > 84 *m*/*z* (decomposition potential 41 V, collision energy 21 Ev) and GABA 104 *m*/*z* > 87 *m*/*z* (decomposition potential 46 V, collision energy 15 eV). A standard curve of GABA and glutamate (SIGMA) in LCA was used in order to determine the concentration of these samples with the Analyst program, 1.6.3. from AB Sciex.

### 4.7. Statistical Analysis

Results are expressed as mean ± standard error. All statistical analyses were performed using software program GraphPad Prism. Normality was assessed using the D’Agostino and Pearson Omnibus, and the Shapiro–Wilk normality tests. Differences in the variances of normally distributed data were assessed using Bartlett’s test. Data with the same variance across groups were analyzed with parametric one-way analysis of variance (ANOVA) followed by Tukey’s post hoc test when there were more than two groups. Data with a different variance across groups were analyzed using the nonparametric Kruskal–Wallis test followed by Dunnett’s post hoc test. A confidence level of 95% was accepted as significant. The number of rats used for each parameter and the statistical procedure used in each case are indicated in the corresponding figure legend.

## 5. Conclusions

This report identified several mechanisms by which the neuroinflammation-induced increase in BDNF enhances GABAergic neurotransmission in the cerebellum of hyperammonemic rats. Hyperammonemia-induced changes in GAD65 and GAD67, and in the membrane expression of GAT3 and of the γ2, α2, and β3 subunits of GABA_A_ receptors were a consequence of the enhanced activation of TrkB via the increased levels of BDNF (Figure 10). Moreover, these steps of GABAergic neurotransmission could be normalized by blocking the TNFR1–SP1PR2–CCL2–CCR2–BDNF–TrkB pathway at any of its steps. This allows for improving motor and cognitive function in patients with hepatic encephalopathy and likely also patients with other pathologies associated with neuroinflammation-induced alterations in GABAergic neurotransmission, such as autism [64], Huntington’s disease [65], Alzheimer’s disease [66,67], Parkinson’s disease, and multiple sclerosis [68], opening new therapeutic possibilities to improve motor and cognitive function in these patients.

## Data Availability

All raw data used and analyzed for the current study are available from the corresponding author on reasonable request.

## Abbreviations

CEBA	Comite de Experimentación y Bienestar animal
CCL2	Monocyte chemoattractant protein-1, MCP-1
CCR2	C–C chemokine receptor type 2
HE	Hepatic encephalopathy
HPLC-MS	Liquid chromatography-mass spectrometry
MHE	minimal hepatic encephalopathy
S1PR2	Sphingosine-1-phosphate receptor 2
TNFR1	Tumor necrosis factor receptor 1
